# Association between Asthma and Autism Spectrum Disorder: A Meta-Analysis

**DOI:** 10.1371/journal.pone.0156662

**Published:** 2016-06-03

**Authors:** Zhen Zheng, Li Zhang, Tingting Zhu, Jichong Huang, Yi Qu, Dezhi Mu

**Affiliations:** 1 Department of Pediatrics, West China Second University Hospital, Sichuan University, Chengdu 610041, China; 2 Key Laboratory of Obstetric & Gynecologic and Pediatric Diseases and Birth Defects of Ministry of Education, Sichuan University, Chengdu 610041, China; 3 Department of Pediatrics and Neurology, University of California San Francisco, San Francisco, CA 94143, United States of America; National Cancer Center, JAPAN

## Abstract

**Objective:**

We conducted a meta-analysis to summarize the evidence from epidemiological studies of the association between asthma and autism spectrum disorder (ASD).

**Methods:**

A literature search was conducted using PubMed, Embase, and Cochrane library for studies published before February 2nd, 2016. Observational studies investigating the association between asthma and ASD were included. A random effects model was used to calculate the pooled risk estimates for the outcome. Subgroup analysis was used to explore potential sources of heterogeneity and publication bias was estimated using Begg's and Egger's tests.

**Results:**

Ten studies encompassing 175,406 participants and 8,809 cases of ASD were included in this meta-analysis. In the cross-sectional studies, the prevalence of asthma in ASD was 20.4%, while the prevalence of asthma in controls was 15.4% (*P* < 0.001). The pooled odds ratio (OR) for the prevalence of asthma in ASD in the cross-sectional studies was 1.26 (95% confidence interval (CI): 0.98–1.61) (*P* = 0.07), with moderate heterogeneity (I^2^ = 65.0%, *P* = 0.02) across studies. In the case-control studies, the pooled OR for the prevalence of asthma in ASD was 0.98 (95% CI: 0.68–1.43) (*P* = 0.94), and there was no evidence of an association between asthma and ASD. No evidence of significant publication bias on the association between asthma and ASD was found.

**Conclusions:**

In conclusion, the results of this meta-analysis do not suggest an association between asthma and ASD. Further prospective studies ascertaining the association between asthma and ASD are warranted.

## Introduction

Autism spectrum disorder (ASD) is a neurodevelopmental disorder categorized by abnormalities in social interaction, impairment in language and communication, restrictive or repetitive interests, and stereotyped behaviors and movements [1]. The prevalence of ASD is from 4 to 60-100/10,000 with a male-to-female ratio of 3–4:1 over the last decade [2]. These conditions begin in early childhood and persist for the rest of the individual’s life, which increases healthcare costs and the financial burden to both family and society. However, the etiology of ASD remains unclear. Evidence shows that dysregulated immune and inflammatory processes might be involved in the pathogenesis of ASD [3, 4]. Studies have found evidence of many immune-mediated conditions being frequently comorbid in autism [5, 6, 7, 8]. Akintunde et al. [7] found a higher prevalence of asthma in children with ASD compared to controls (26.7% vs. 7.3%). Prevalence of rhinitis was also higher among ASD cases compared to controls (16% vs. 12.9%) [8]. Food allergy prevalence was reported 0.9% in children with ASD while 0.5% in controls [8].

Asthma is one of the most common chronic respiratory diseases with an increasing prevalence and financial burden all over the world [9]. Asthma is an immune-mediated disorder categorized by an inappropriate inflammatory response in the lungs. Nowadays, ASD has been described imbalance in immune and inflammatory processes. Altered levels of cytokines, inflammatory markers and immunoglobulin have been found in ASD [10]. It seems more likely that asthma and ASD could arise from common or related pathologic disruptions of the immune system [11, 12]. The association between asthma and ASD has been increasingly recognized. However, the results were conflicting. Chen et al. [6] reported that the prevalence of asthma in ASD was higher than that in controls, while Jyonouchi et al. [13] reported a negative association between them. Recently, more relevant studies have been published [7, 14,15], allowing for the analysis of the relationship between these two disorders.

Many publications are rising a problem of diagnostic difficulties in recognizing chronic diseases if they coexist with ASD [16]. Medical comorbidities like allergies in the general population are often related to increased irritability and poorer functional outcomes in ASD [16]. In addition, it has been showed that ASD with asthma are more likely to experience depression, poorer parenting and competing demands [17]. Thus, if ASD had a higher prevalence of asthma, this association will promote more active strategies to timely diagnose and manage asthma to avoid adverse outcomes in ASD. Accordingly, examining the association between asthma and ASD from an epidemiologic perspective may promote efforts to implement preventive public health strategies in this subpopulation. Therefore, the aim of this study was to systematically review the prevalence of asthma among people with ASD compared to people without ASD.

## Materials and Methods

### Literature search

Two authors searched PubMed, Embase and Cochrane library for relevant articles published before February 2nd, 2016 using both Medical Subject Heading (MeSH) terms and the free text terms: [“ASD” OR “autism spectrum disorder” OR “autistic disorder” OR “Asperger syndrome” OR “autism”] and [“asthma” OR “bronchial asthma” OR “wheeze” OR “wheezing”]. In addition, the references of the included articles and previous meta-analyses were searched manually to identify additional studies.

We restricted the search to human studies published in English. The titles and abstracts of the retrieved studies were reviewed to exclude studies that were clearly irrelevant. Then, two authors independently read the full text of the remaining studies to assess their eligibility according to the inclusion criteria. Disagreements about the inclusion/exclusion of a study were resolved by a third author, who independently examined the studies, and consensus was reached.

### Study Selection

Studies were eligible for analysis if they met all of the following criteria: (1) they were about the association between asthma and ASD; (2) they were case-control or cohort studies or cross-sectional studies; and (3) they provided the raw data or odds ratio (OR) with associated 95% confidence interval (CI).

Exclusion criteria for the study were as follows: (1) reviews, case reports, case-only studies, animal studies, simple commentaries; and (2) overlapped publications.

### Data extraction

Two authors extracted data from the included articles, with particular regard to: first author’s name, publication year, country of region, defect type, study design, number of cases and controls, the percentage of male, measurements of ASD and asthma, mean age, and adjusted confounders. If participants overlapped between studies, the one with largest sample size was included in the meta-analysis.

### Quality evaluation

Two authors independently assessed the quality of each included study using the Newcastle-Ottawa Quality Assessment Scale (NOS) for the studies to determine the quality of selection, comparability, exposure, and outcome of study participants, with a maximum of 9 points. We divided the study quality into three categories: (1) high quality (scored 7–9); (2) moderate quality (scored 4–6); and (3) low quality (scored 0–3). Disagreements were resolved through mutual discussion.

### Statistical Analysis

ORs were used to assess the association between asthma and ASD. We pooled the ORs across studies using the Mantel-Haenszel formula (fixed-effect model) or the DerSimonian-Laird formula (random-effect model). A fixed-effect model was chosen when low heterogeneity existed; otherwise, a random-effect model was adopted. Heterogeneity across the studies was tested using the I^2^ and Q statistic, which is a quantitative measure of inconsistency across studies, with suggested thresholds for low (25%-50%), moderate (50%-75%) and high (> 75%) heterogeneity. The Q statistic was considered significant if *P* < 0.1, and I^2^ > 50% indicated high heterogeneity.

Potential publication bias was assessed by visual inspection of the funnel plot. Begg's and Egger's tests were used to estimate the severity of publication bias, with *P* < 0.05 considered statistically significant. We conducted subgroup analyses in studies to examine the source of potential heterogeneity based on condition (ASD or autism or Asperger’s syndrome), geographic location (America or Europe or Asia) and case number (≤ 100 or > 100).

We carried out the sensitivity analysis by excluding results which the quality assessment of the studies were below the average score. A forest plot was used to show the ORs and 95% CIs for each study, as well as the pooled ORs and 95% CIs. Statistical analysis was performed using Stata 12.0 (Stata Corp, College Station, Texas, USA) and Cochrane Collaboration Review Manager 5.1.2 (Cochrane Collaboration, Oxford, UK) software.

## Results

### Literature search

**Fig 1:**
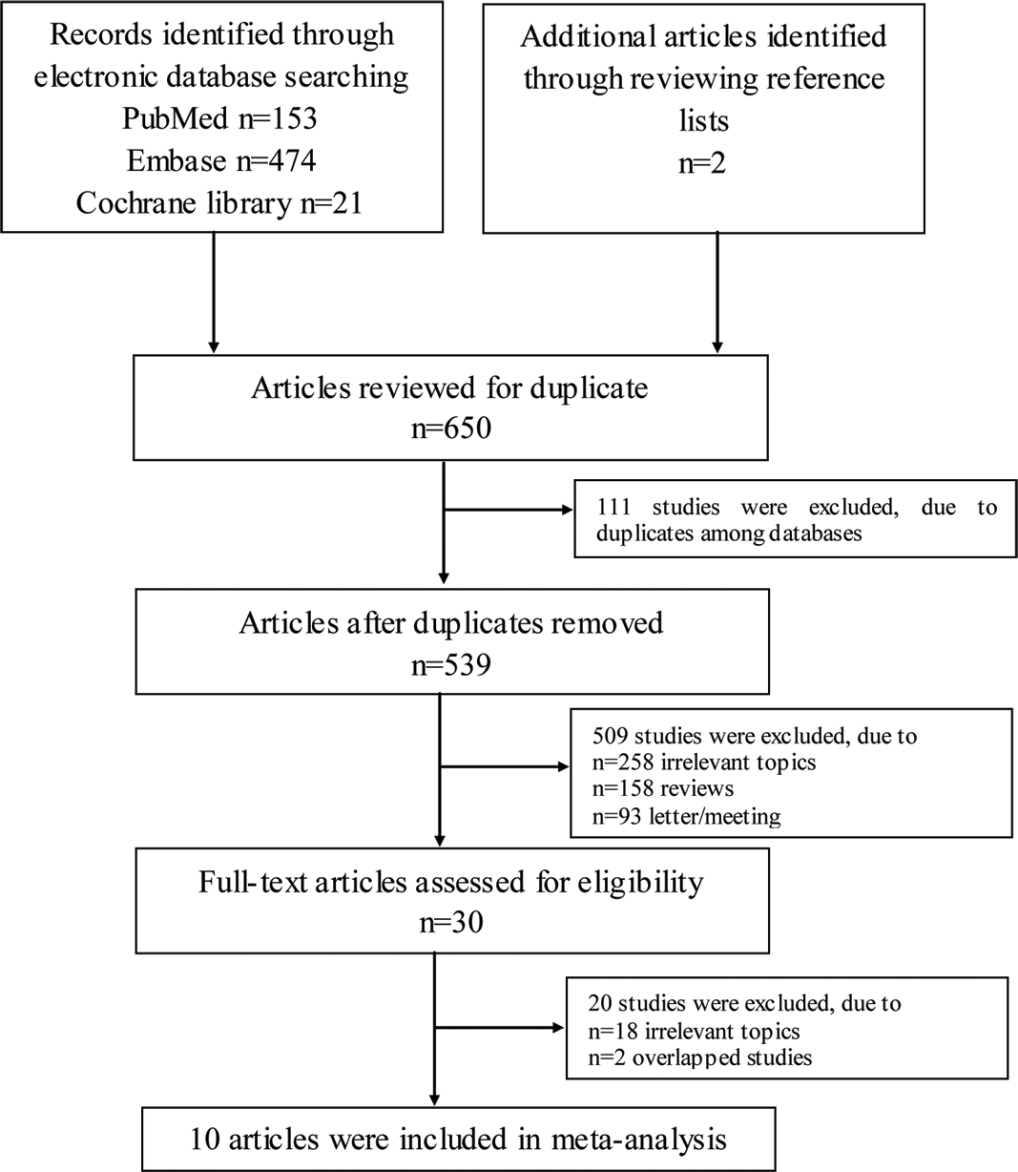
The detailed steps of the literature search.

A total of 650 citations were yielded in the initial search, with 153 from PubMed, 474 from Embase, 21 from Cochrane library and two from reviewing references. After excluding 111 duplicate studies, 258 with irrelevant topics, 158 reviews and 93 letters/meetings, 30 papers were identified on asthma and ASD and were subjected to a detailed evaluation. Subsequently, eighteen studies were excluded because of irrelevant outcomes. Two reports were excluded due to overlapping. Finally, ten studies fulfilled all the inclusion criteria, including 175,406 participants and 8,809 cases of ASD in this meta-analysis. A detailed flow chart of the search and selection process is presented in Fig 1.

### Study Characteristics

The characteristics of the ten selected studies are presented in Table 1. All of the studies were published between 2008 and 2015. Of the ten included studies, there were five case-control studies [7, 8, 13, 14, 18] and five cross-sectional studies [15, 19–22], with a total of 175,406 participants and 8,809 cases of ASD involved. Six studies were conducted in the United States [7, 8, 13, 18, 20, 22], one in Turkey [19], one in Brazil [21], one in Poland [14], and one in China [15]. The sample sizes varied widely, ranging from 15 [21] to 5,565 [8] ASD individuals and from 30 [21] to 76,545 [20] controls. Similarly, the mean age of ASD and control individuals varied broadly, ranging from 2 to 26 years old.

**Table 1:** Characteristics of studies on the association between asthma and ASD.

First author, year	Country	Condition	Study design	Case/control	Case/control Male(%)	ASD diagnostic criteria	Asthma diagnostic criteria	Mean age	Adjusted confounders
Bakkaloglu 2008	Turkey	Autism	Cross-section	30/39	nr	DSM-IV	Self-report	2–4	age, gender
Schieve 2012	USA	Autism	Cross-section	375/35775	77.9/48.8	Self-report	Self-report	3–17	age, gender, race/ethnicity, maternal education
Kotey 2014	USA	Autism	Cross-section	1412/76545	77.9/51.5	Self-report	Self-report	≥3and<18	age, gender, BMI, race, history of brain injury, exposure to secondhand smoke, socio-economic status
Magalhães 2009	Brazil	Asperger's syndrome	Cross-section	15/30	nr	DSM-IV-TR, Filipek's review criteria	Clinical evaluation	7–18	age, gender, socio-economic status
Zerbo 2015	USA	ASD	Case-control	5565/27825	82/82	ICD-9-CM	ICD-9	3–26	age, gender, total membership months
Akintunde 2015	USA	ASD	Case-control	45/69	82.2/87	DSM-IV, ADOS, ADI-R	Self-report	3.57	age, gender
Mrożek-Budzyn 2013	Poland	Autism	Case-control	96/192	93.8/94.2	ICD-10	Self-report	≤14	age, gender, physician's practice
Lin 2014	China	ASD	Cross-section	578/25688	81/79.4	ICD-9-CM	ICD-9-CM	<18	age, gender, level of urbanization
Jyonouchi 2008	USA	ASD	Case-control	133/43	88/72.1	DSM-IV, ADI-R, ADOS	NIH guideline criteria	3.3–12.5	age, gender
Lyall 2015	USA	ASD	Case-control	560/391	86/83	ADI-R, ADOS	Self-report	2–5	age, gender, geographic area, child recurrent infections in early life, maternal smoking

Abbreviation: nr = no reported.

For ASD diagnostic criteria, two were self-reported [20, 22], and the others were reported according to the Diagnostic and Statistical Manual (DSM-IV) [7, 13, 19], International Classification of Diseases, 9th Revision, Clinical Modification (ICD-9-CM) [8, 15], International Classification of Diseases, 10th Revision (ICD-10) [14], Autism Diagnostic Interview-Revised (ADI-R) [7, 13, 18], Autism Diagnostic Observation Schedule-Generic (ADOS) [7, 13, 18], DSM-IV-TR and Filipek's review criteria [21]. For the diagnostic criteria of asthma, six studies were self-reported [7, 14, 18–20, 22], one was measured according to ICD-9 [8], one was diagnosed by ICD-9-CM [15], one was determined by NIH guideline criteria [13], and one was measured by clinical evaluation [21].

### The prevalance of asthma in ASD

**Fig 2:**
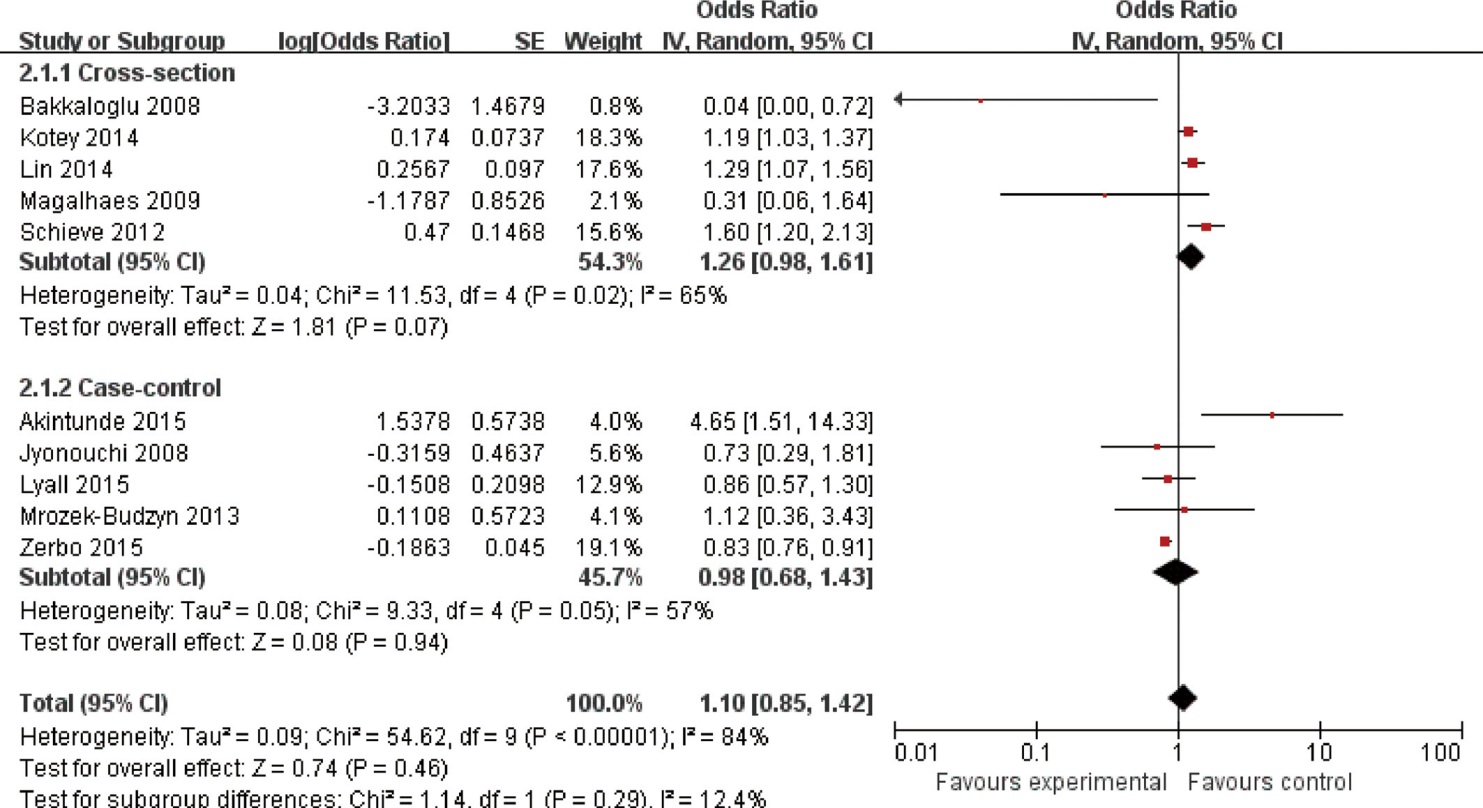
Forest plot of the association between asthma and ASD.

In the cross-sectional studies, the prevalence of asthma in ASD was 20.4%, while the prevalence of asthma in controls was 15.4% (*P* < 0.001). The pooled OR for the prevalence of asthma in ASD was 1.26 (95% CI: 0.98–1.61) (*P* = 0.07), with moderate heterogeneity (I^2^ = 65.0%, *P* = 0.02) in the cross-sectional studies (Fig 2).

In the case-control studies, the pooled OR for the prevalence of asthma in ASD was 0.98 (95% CI: 0.68–1.43) (*P* = 0.94), with moderate heterogeneity (I^2^ = 57.0%, *P* = 0.05) across studies (Fig 2).

### Quality evaluation

The results of the quality assessment of the included studies are shown in Table 2. Nine studies were of high quality and one study was of moderate quality. The average score was 7.0 in the cross-sectional studies and the average score was 8.0 in the case-control studies.

**Table 2:** Quality assessment of the included studies by the Newcastle–Ottawa Scale.

First author, year	Study design	Selection	Comparability	Exposure/Outcome	Total scores
Bakkaloglu 2008	Cross-section	★★★	★★	★★	7
Schieve 2012	Cross-section	★★★	★★	★	6
Kotey 2014	Cross-section	★★★	★★	★★	7
Magalhães 2009	Cross-section	★★★	★★	★★	7
Zerbo 2015	Case-control	★★★	★★	★★	7
Akintunde 2015	Case-control	★★★★	★★	★★★	9
Mrożek-Budzyn 2013	Case-control	★★★★	★★	★★★	9
Lin 2014	Cross-section	★★★★	★★	★★	8
Jyonouchi 2008	Case-control	★★★	★★	★★	7
Lyall 2015	Case-control	★★★★	★★	★★	8

### Publication bias

**Fig 3:**
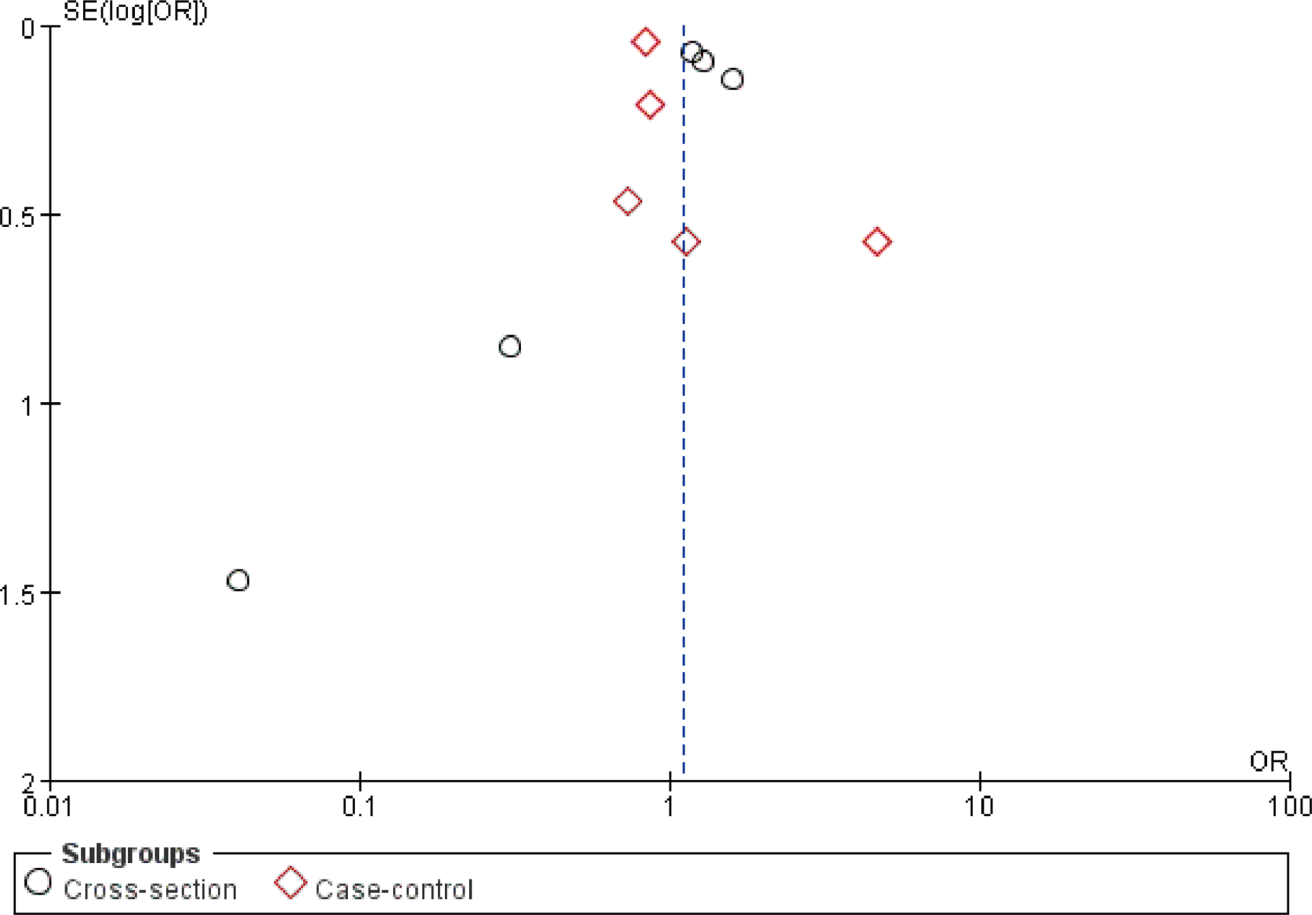
Funnel plot of the association between asthma and ASD.

Visual inspection of the funnel plot indicated the potential publication bias (Fig 3). However, Begg's and Egger's tests did not show significant evidence of publication bias among the included cross-sectional studies (Begg's test, *P* = 0.462; Egger's test, *P* = 0.270) and the case-control studies (Begg's test, *P* = 0.086; Egger's test, *P* = 0.313).

### Subgroup analysis and sensitivity analysis

Table 3 presents the results of subgroup analysis stratified by condition, geographic location and case number. In subgroup analysis by condition, geographic location and case number, the subgroup differences was not statistically significant except the subgroup analysis by case number in the cross-sectional studies.

**Table 3:** Summary results of association between asthma and ASD.

Variables	No. of studies	*I2%*	Pheterogeneity	OR(95%CI)
**Cross-section**	**5**	**65**	**<0.05**	**1.26(0.98,1.61)**
**Condition**				
ASD	1	Not applicable	Not applicable	1.29(1.07,1.56)
autism	3	77	<0.05	1.26(0.81,1.96)
Asperger's syndrome	1	Not applicable	Not applicable	0.31(0.06,1.64)
**Geographic locations**				
America	3	67	0.05	1.28(0.90,1.80)
Europe	1	Not applicable	Not applicable	0.04(0.00,0.72)
Asia	1	Not applicable	Not applicable	1.29(1.07,1.56)
**Case number**				
≤100	2	30	>0.05	0.16(0.02,1.02)
>100	3	39	>0.05	1.30(1.12,1.50)
**Case-control**	**5**	**57**	**0.05**	**0.98(0.68,1.43)**
**Condition**				
ASD	4	67	<0.05	0.99(0.65,1.51)
autism	1	Not applicable	Not applicable	1.12(0.36,3.43)
**Geographic locations**				
America	4	67	<0.05	0.99(0.65,1.51)
Europe	1	Not applicable	Not applicable	1.12(0.36,3.43)
**Case number**				
≤100	2	68	>0.05	2.28(0.56,9.23)
>100	3	0	>0.05	0.83(0.76,0.90)

We carried out the sensitivity analysis by excluding results which the quality assessment of the studies were below the average score. The pooled OR was 1.15 (95% CI: 0.86–1.54) (*P* = 0.36) when excluded the study by Schieve et al. in the cross-sectional studies. The pooled OR was 1.51 (95%CI: 0.56–4.04) (*P* = 0.42) when excluded the studies by Jyonouchi et al. and Zerbo et al. in the case-control studies.

## Discussion

The results of meta-analysis of ten studies with a total of 175,406 participants produced by the random-effects model do not suggest an association between asthma and ASD.

The hypothesis of association between asthma and ASD is plausible. Asthma is a complex and multifaceted illness characterized by chronic inflammation [23]. Nowadays, inflammatory pathways activated due to immune dysfunction, have been proposed as possible mechanisms implicated in the pathogenesis of ASD, which suggests asthma and ASD have common etiologies [11, 12].

Altered levels of cytokines, inflammatory markers and immunoglobulin have been found in ASD [10]. Recently, studies on asthma indicated increased serum levels of pro-inflammatory cytokines such as IL-12, IL-13, IL-17 in individuals with asthma [24, 25], which were elevated in patients with ASD as well [7, 11, 26, 27]. In addition, inflammatory markers, such as mast cells, play important roles in the pathophysiology of asthma [24]. Similarly, increased mast cells have also been found in ASD children [28]. The immunoglobulin imbalances are important in the pathogenesis of asthma. Studies of immune function in individuals with ASD have also found immunoglobulin imbalances, including increased levels of plasma IgG4, reduced levels of IgM or of total IgG [29, 30, 31], which further suggests a potential link between asthma and ASD. Furthermore, there are studies showed that maternal autoimmune conditions, asthma, allergies and prematurity were associated with having an ASD child [32, 33, 34]. Croen et al. [32] reported that more than 2-fold elevated risk of having an ASD child was observed in maternal asthma and allergy diagnoses recorded during the second trimester of pregnancy, which further demonstrated the association between asthma and ASD. But our meta-analysis do not suggest an association between asthma and ASD. Further studies are needed to confirm this association between asthma and ASD.

Our findings presented some advantages. First, this is the first comprehensive meta-analysis conducted to assess the association between asthma and ASD. Second, a large number of cases and participants were included, allowing a much greater possibility of reaching reasonable conclusions between asthma and ASD. Third, the sensitivity analysis did not materially alter the final results, which increased the robustness of our findings. We did not detect a significant publication bias, suggesting that our results are reliable.

However, there are some limitations to our meta-analysis. The first is the potential misclassification of asthma and ASD status of participating children, which relied on self-reported questionnaires to diagnose ASD and asthma. Undiagnosed cases of ASD might have occurred if parents lacked access to healthcare for confirmation of diagnosis [25]. Also, misdiagnosis by clinicians might have led to the misclassification of ASD. Similarly, the misclassification of asthma might have occurred if the parents perceived a common upper respiratory tract infection as asthma, which contributed to the increased risk of asthma in controls. Future work should attempt to adopt standardized diagnostic approaches for the selection of participants and assessment of asthma which conducted by trained clinicians. Furthermore, due to the impaired expressive language, aberrant behaviors and lower tolerance to diagnostic measures, diagnosing asthma may be more challenging in ASD [4]. Thus, asthma tends to be under-diagnosed in patients with ASD because symptoms might be masked by behavioral problems [16]. Additionally, there was moderate heterogeneity in the analysis, which may weaken the strength of our findings. Although the subgroup analysis by case number in the cross-sectional studies may partly explain the heterogeneity. Residual confounding factors across studies remain as a cause for concern in this meta-analysis. Therefore, heterogeneity was still an inevitable problem that may affect the precision of the overall results. Thirdly, the included studies in this meta-analysis were case-control and cross-sectional studies, which lacked of information on timing, precluding ability to identify a temporal or causal relationship between asthma and ASD. Thus, further studies with a prospective design are needed to confirm this association between asthma and ASD. Fourthly, other limitations such as confounding factors including the use of medication, the clinical severity, family history, personal lifestyle, and environmental factors were not adjusted, the influence of these factors were not controlled.

In conclusion, the results of this meta-analysis do not suggest an association between asthma and ASD. Further prospective studies by using standardized diagnostic approaches for the selection of participants and assessment of asthma to ascertain the association between asthma and ASD are warranted.

## Supporting Information

S1 File PRISMA checklist. (DOC)
